# How Does Passive Cyber Incivility Influence Work Engagement? A Serial Mediation via Motivation and Emotion

**DOI:** 10.3390/bs15020113

**Published:** 2025-01-22

**Authors:** Yi Lu, Yu Yan, Shuai-Ping Xiao, Kai-Chen Zhao, Zhao-Xue Cao, Yan-Hui Zhou

**Affiliations:** 1 Academy of Advanced Interdisciplinary Studies, Wuhan University, Wuhan 430072, China; 2 School of Marxism, Hubei University of Economics, Wuhan 430205, China

**Keywords:** passive cyber incivility, work engagement, stress mindset, intrinsic motivation, emotional exhaustion

## Abstract

Passive cyber incivility, with its ambiguity and offensive nature, can have a detrimental impact on employees’ well-being and negative work consequences. To explore passive cyber incivility in depth, we examined its effects through both motivational and affective channels. Over the course of a month, this study conducted a three-wave survey across industries, involving a sample of 306 employees. Results showed that passive cyber incivility has no direct impact on work engagement. Instead, it hampered work engagement through the serial mediation of intrinsic motivation and emotional exhaustion. Overall, this study aims not only to raise awareness of the risks associated with passive cyber incivility but also to contribute to the existing literature on work engagement by investigating the mediating processes that have not been sufficiently studied.

## 1. Introduction

In recent years, the shift to the digital world has revolutionized the way workers communicate with each other, removing the boundaries of time and place. However, research shows that over 90% of employees have experienced uncivil online behavior in the workplace (Xiao et al., 2023). Cyber incivility encompasses a wide spectrum of behaviors, including both active and passive forms of disrespect in digital communication (Giumetti et al., 2012). Although the existing literature has focused on active cyber incivility, like hostile language or openly rude comments, passive cyber incivility (PCI) has not received as much attention. PCI refers to subtle and unclear behaviors, like ignoring messages or handling sensitive topics in a cold, impersonal way (Lim & Teo, 2009). Unlike the active form of incivility, the ambiguity of PCI forces employees to spend extra mental energy trying to figure out the intent behind it (Zhou et al., 2022). To make matters worse, PCI is often not easily detected or ignored, yet it can still damage workplace morale and employee engagement over time.

The insidious nature of PCI makes it difficult to identify. Yuan et al. (2020) argued that PCI is closely associated with unclear or ambiguous appraisals. Zhou et al. (2022) suggested that PCI may trigger negative affect, leading to cyberloafing. Although PCI is less obvious than the active form, it still has a serious impact on employees’ psychological well-being and organizational outcomes (Torres et al., 2024). Furthermore, PCI has become an increasingly common phenomenon due to the deeper integration of technology and interpersonal interactions in the workplace. Research highlights how constant connectivity through emails and messaging platforms has deepened our dependence on digital communication, blurring the lines of traditional norms and professional conduct (Bartlett & Bartlett, 2016). The growing dependence on asynchronous communication (e.g., email and instant messaging) in remote work settings has exacerbated the prevalence of PCI, particularly during the COVID-19 pandemic. Therefore, it is vital to address PCI as a distinct issue, rather than conflating it with other forms of online incivility or disrespectful behavior.

PCI not only impacts an individual’s well-being but also significantly threatens work engagement, defined as the positive mental state marked by vigor, dedication, and absorption in occupational activities (Chen et al., 2020). Engaged employees display high levels of energy and intrinsic motivation, crucial for optimal job performance. However, the stress induced by PCI can lead to decreased work engagement, as employees struggle to maintain focus and commitment in the face of subtle disrespect. Addressing PCI requires substantial resources, which can detract from an employee’s capacity to remain fully engaged with their work.

This study explores how PCI affects work engagement by examining intrinsic motivation and emotional exhaustion as key factors. Intrinsic motivation refers to the inner drive to perform tasks because they are inherently satisfying, while emotional exhaustion is the feeling of being emotionally drained due to ongoing stress (Chen et al., 2020; Fishbach & Woolley, 2022). As stated by the Conservation of Resources (COR) theory, people strive to gain and safeguard resources that are essential for managing workplace demands (Hobfoll, 1989). However, PCI, as a subtle and ambiguous stressor, depletes these resources, reducing intrinsic motivation and increasing emotional exhaustion. Its unclear nature further amplifies this resource loss, as employees must invest additional psychological and emotional energy to interpret its intent and cope with its effects (Hobfoll et al., 2018). By focusing on these two mediators, this study aims to uncover the specific pathways through which PCI undermines work engagement.

In addition, this study also considers stress mindset as a moderator in relationships. Stress mindset reflects an individual’s belief about the nature of stress, with a positive stress mindset seeing stress as an opportunity for growth, and a negative stress mindset viewing it as harmful (Crum et al., 2023). We propose that this mindset affects how employees perceive and react to PCI, thereby potentially buffering or exacerbating perceptions of PCI, which in turn affects work engagement. By integrating stress mindset into the analysis, this study seeks to offer a more nuanced understanding of individual differences in response to PCI.

While past studies have investigated cyber incivility as a broader construct, few have delved into the nuanced dynamics of PCI as a distinct and insidious form of workplace disrespect. This study addresses this gap by the following: (1) Investigating PCI as a distinct construct and exploring its effects on work engagement; (2) examining intrinsic motivation and emotional exhaustion as dual mediators, offering new insights into the resource-based mechanisms through which PCI impacts work engagement (Fishbach & Woolley, 2022); (3) introducing stress mindset as a moderator, acknowledging individual differences in stress perception and response, and providing a nuanced understanding of how employees manage PCI’s impacts (Crum et al., 2017). Through these contributions, this study aims to shed light on how PCI affects workplace dynamics and offer practical strategies to reduce its harmful effects on employee well-being and performance.

## 2. Literature Review

### 2.1. Passive Cyber Incivility and Work Engagement

Passive cyber incivility refers to subtle forms of inappropriate online behavior, including behaviors such as neglecting to respond to messages in the workplace (Tasoulis et al., 2023). In contrast to overt forms of cyber incivility such as shouting in written text or embedding sarcastic, mean, or rude language, PCI, while more subtle in nature, nonetheless conveys a significant lack of respect (Zhou et al., 2022).

Work engagement represents the level of effort, enthusiasm, and energy an individual dedicates to their work (Chen et al., 2020). Incivility can have a significant negative impact on an individual’s psychological state, which usually manifests itself in decreased concentration and excessive energy expenditure (Schaufeli & Bakker, 2010). Research has consistently shown a significant negative correlation between workplace incivility and employee engagement (Chen et al., 2020). PCI, a form of incivility, may exacerbate this negative impact due to its indirect and ambiguous nature (Wolter et al., 2023). Although individual studies have suggested that certain uncivil behaviors may motivate employees to temporarily increase their work engagement through compensatory efforts in the short term, the overall effect of PCI on work engagement tends to weaken in the long term.

According to the COR theory (Hobfoll, 1989), PCI could be regarded as a stressor that consumes psychological resources. Employees affected by PCI often need to exert extra emotional effort to understand the ambiguous intentions behind the behavior. This extra effort can exacerbate resource depletion, leading to low energy, distraction, and reduced commitment levels (Hobfoll et al., 2018). At the same time, the passive nature of PCI further increases employees’ anxiety and uncertainty, impeding their focus on the task, and thus negatively affecting work engagement (Ugwu et al., 2023).

Therefore, we propose that PCI primarily leads to reduced work engagement due to its resource-depleting nature.

Thus, we hypothesized the following:

**H1.**  *Passive cyber incivility negatively predicts work engagement.*

### 2.2. The Mediating Role of Intrinsic Motivation

Intrinsic motivation illustrates the personal satisfaction and sense of accomplishment an individual derives from engaging in job tasks that provide a sense of challenge and foster competence (Ryan & Deci, 2020). Intrinsic motivation is closely tied to satisfying three fundamental psychological needs: competence, autonomy, and relatedness. When these needs are satisfied, intrinsic motivation flourishes, leading employees to engage in tasks with enthusiasm, persistence, and creativity, regardless of extrinsic rewards (Shin et al., 2019). However, when these needs are thwarted, intrinsic motivation is likely to decline, adversely affecting job-related outcomes such as work engagement.

PCI disrupts the fulfillment of these psychological needs, thereby undermining intrinsic motivation. PCI, characterized by subtle disrespectful behaviors such as resorting to impersonal communication in sensitive situations, can erode employees’ sense of competence, as they may feel undervalued or incapable of managing workplace interactions. Similarly, PCI threatens autonomy, as employees may perceive it as a lack of respect for their time, decisions, or priorities. Finally, PCI hinders relatedness, as it diminishes the sense of interpersonal connection and mutual respect critical to workplace relationships.

From the perspective of COR theory (Hobfoll et al., 2018), PCI acts in a way that depletes employees’ resources—both emotional and cognitive—that are necessary to sustain intrinsic motivation. The ambiguity of PCI demands additional cognitive effort to interpret its intent and navigate its effects, leaving employees with fewer resources to focus on tasks that bring intrinsic satisfaction. This resource depletion can also trigger avoidance or procrastination behaviors as coping mechanisms, further reducing motivation (Hobfoll et al., 2018). In extreme cases, employees may intentionally keep their intrinsic motivation low as a resource preservation strategy, prioritizing survival over engagement (Law et al., 2010).

On the other hand, intrinsic motivation is an essential driver of work engagement (Schaufeli & Bakker, 2010). Employees with high intrinsic motivation tend to invest additional effort in their work, thereby enhancing both their performance and organizational outcomes (Rode, 2016). However, as PCI drains the resources required to sustain motivation, employees may lose interest in their tasks, experience diminished job satisfaction, and withdraw from work. Consequently, intrinsic motivation serves as a critical mechanism through which PCI undermines work engagement (Sawhney & Michel, 2022).

Thus, based on COR theory, we suggest that intrinsic motivation mediates the relationship between PCI and work engagement, as PCI undermines the psychological needs essential for intrinsic motivation and depletes the resources needed to sustain it. Employees with lower intrinsic motivation are less likely to exhibit the facets of work engagement, making intrinsic motivation a key pathway through which PCI exerts its negative effects.

Consequently, we propose Hypothesis 2:

**H2.**  *Intrinsic motivation acts as a mediator between passive cyber incivility and work engagement.*

### 2.3. The Mediating Role of Emotional Exhaustion

Emotional exhaustion is a state of emotional and physical fatigue caused by insufficient recovery opportunities (Chen et al., 2020). This exhaustion occurs due to the depletion of emotional resources, often resulting from high work stress, ultimately impacting both employee well-being and performance (Esbati & Korunka, 2021). Among the three dimensions of burnout—emotional exhaustion, depersonalization, and low personal accomplishment—emotional exhaustion is the most immediate and fundamental response to stress, making it particularly relevant for examining the effects of PCI through the lens of COR theory (Maslach et al., 2001). By focusing on emotional exhaustion as a mediating variable, we aim to capture the more immediate and proximal impacts of PCI, whereas depersonalization and low personal accomplishment are typically downstream consequences of prolonged exposure to stressors.

PCI, characterized by behaviors like ignoring emails, failing to acknowledge contributions, or displaying discourtesy in online communications, can foster feelings of disrespect and isolation among employees (Xiao et al., 2023). According to COR theory, PCIs significantly drain employees’ psychological and emotional resources, thus triggering stress responses. This lack of recognition prompts cognitive rumination and intensifies emotional depletion, which further intensifies emotional depletion. Additionally, ambiguous responses or criticisms stemming from PCI can exacerbate anxiety and frustration, accelerating resource depletion (Lazarus, 1991).

Emotional exhaustion undermines work engagement (Schaufeli & Bakker, 2010). Employees with emotional exhaustion struggle to maintain cognitive flexibility, focus, and energy, making it difficult to stay engaged with their work. To conserve their remaining resources, they may instinctively withdraw from tasks, resulting in lower levels of vigor and dedication. PCI, through its resource-depleting effects, disrupts work engagement by exhausting employees’ psychological capacity to remain motivated and committed. Unlike depersonalization or low personal accomplishment, emotional exhaustion captures the immediate strain caused by PCI, making it a central mechanism through which PCI impacts work engagement.

Therefore, emotional exhaustion is a key mechanism through which PCI affects work engagement. PCI depletes employees’ emotional and psychological resources, reducing their energy and dedication to work. Building on this, we suggest the following hypothesis:

**H3.**  *Emotional exhaustion acts as a mediator between passive cyber incivility and work engagement.*

### 2.4. The Serial Mediation Role from Intrinsic Motivation to Emotional Exhaustion

Emotional exhaustion and intrinsic motivation are key mediators in the relationship between PCI and work engagement. Exposure to PCI disrupts employees’ psychological resources, as explained by COR theory (Hobfoll, 1989). PCI undermines intrinsic motivation by obstructing the fulfillment of basic psychological needs, such as competence, autonomy, and relatedness, which are critical for sustaining an individual’s intrinsic satisfaction and drive toward work tasks (Ryan & Deci, 2020).

The sequential relationship between intrinsic motivation and emotional exhaustion can be explained through COR theory’s resource loss spiral (Hobfoll et al., 2018). When intrinsic motivation is diminished due to PCI, employees must draw from their remaining emotional reserves to maintain productivity and meet workplace expectations. This additional strain depletes emotional resources more rapidly, intensifying emotional exhaustion. In turn, heightened emotional exhaustion obstructs employees’ ability to remain engaged in their work, as they lack the energy, focus, and psychological resources necessary to maintain vigor and dedication. Work engagement is particularly sensitive to resource depletion (Schaufeli & Bakker, 2010). Exhausted employees are less likely to invest the emotional and cognitive energy required to stay engaged in their roles, leading to withdrawal and reduced performance.

This serial mediation pathway highlights the interconnected roles of intrinsic motivation and emotional exhaustion in the PCI–work engagement relationship. PCI acts as a stressor that undermines intrinsic motivation by draining the psychological resources needed to sustain autonomous drive. The decline in intrinsic motivation imposes additional emotional strain, further depleting resources and heightening emotional exhaustion.

Thus, we hypothesize the following:

**H4.**  *Intrinsic motivation and emotional exhaustion serve as a serial mediation pathway between passive cyber incivility and work engagement. Specifically, PCI reduces intrinsic motivation, which in turn increases emotional exhaustion, leading to lower work engagement.*

### 2.5. Stress Mindset as a Moderator

Stress mindset refers to an individual’s beliefs about the nature of stress, influencing how they respond to stressful situations (Crum et al., 2013). Research shows that stress mindset moderates the impact of stress on emotional and behavioral outcomes. Individuals with a higher stress mindset are more likely to interpret stress positively and experience improved psychological well-being (Huebschmann & Sheets, 2020; Y. Jiang et al., 2019). In contrast, those with a lower stress mindset tend to focus on the negative aspects of stress, leading to higher anxiety and avoidance behaviors. Based on COR theory (Hobfoll, 1989), stress mindset influences how individuals allocate and conserve resources. A higher stress mindset helps protect resources, while a lower stress mindset accelerates resource depletion (Keech et al., 2018).

Intrinsic motivation, driven by the satisfaction of basic psychological needs, plays a key role in employee engagement (Ryan & Deci, 2020). PCI disrupts these needs, reducing intrinsic motivation. However, stress mindset moderates this relationship. Employees with a higher stress mindset are more likely to perceive PCI as a manageable challenge, which helps preserve intrinsic motivation and work engagement. In contrast, individuals with a lower stress mindset tend to regard PCI as an uncontrollable stressor, leading to resource depletion and decreased intrinsic motivation (Huebschmann & Sheets, 2020).

Thus, we hypothesize the following:

**H5a.**  *Stress mindset moderates the relationship between PCI and intrinsic motivation. For example, such that the negative effect of PCI on intrinsic motivation is weaker for employees with a higher stress mindset.*

Emotional exhaustion describes the depletion of emotional and psychological resources caused by prolonged exposure to stress (Maslach et al., 2001). PCI exacerbates resource depletion by fostering rumination, frustration, and anxiety (Xiao et al., 2023). Stress mindset also moderates this process. Employees with higher stress mindset scores are more likely to perceive PCI as a manageable stressor, reducing the emotional toll of ambiguous and disrespectful behaviors, which helps conserve emotional resources and lowers emotional exhaustion. Conversely, employees with lower stress mindset scores tend to view PCI as uncontrollable, leading to greater emotional strain and higher exhaustion (Huebschmann & Sheets, 2020; Hobfoll et al., 2018).

**Figure 1:**
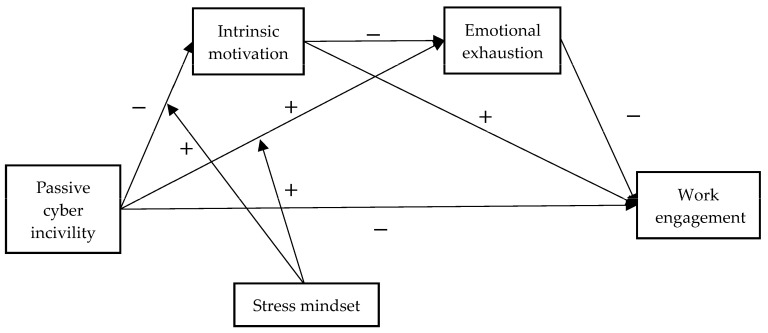
The proposed model. Note: Active cyber incivility is controlled.

Thus, we hypothesize the following:

**H5b.**  *Stress mindset moderates the relationship between passive cyber incivility and emotional exhaustion, such that the positive effect of PCI on emotional exhaustion is weaker for employees with higher stress mindset scores (Figure 1).*

## 3. Methods

### 3.1. Procedure and Participants

The data were obtained via the Credamo questionnaire platform^1^ with a three-wave time-lagged methodology to reduce the risks of common method bias and capture the temporal relationships between variables (T. Jiang & Sedikides, 2022). The sampling method employed was purposive sampling. A total of 306 Chinese employees, representing various professions, including education, manufacturing, and banking, participated in the study. They were guaranteed that their responses would remain anonymous and be used exclusively for scholarly purposes. During the initial phase, we recruited 426 employees, and 327 employees responded in the second stage. Of these eligible participants, 306 data were collected in the final stage. Each phase was 2 weeks apart, and the total time span was 4 weeks. The final effective recovery rate was 71.83%.

Upon providing their consent by signing the consent form, participants were instructed to complete a passive cyber incivility scale, stress mindset scale, and demographic information in the first wave (T1). After that, the participants answered the intrinsic motivation and emotional exhaustion scales in the second wave (T2). In the final wave (T3), participants filled out the work engagement scale. Out of the 306 participants, 55.9% were women. The average age of the participants was 25.20 years, with an average tenure of 7.43 years^2^. Additionally, 90.2% of the participants held a bachelor’s degree.

### 3.2. Measures

The items were rated on a scale from 1 (strongly disagree) to 7 (strongly agree), unless stated otherwise.

*Passive cyber incivility.* The 6-item scale of the Chinese version was revised by Yan and his group (2023), with an example as “Replied to your messages but did not answer your queries”. The items were assessed on a scale from 1 (never) to 5 (always occurs).

*Work Engagement*. To evaluate work engagement, participants were asked to indicate their level of agreement with a 9-item assessment adapted from Gan et al. (2011) in Chinese. An example item is “I feel full of energy at work”.

*Intrinsic Motivation.* Intrinsic motivation was evaluated using the revised Chinese version of the scale from Yang (2017), which was originally adapted from Kuvaas (2006). An example item includes “I find the tasks I perform at work to be enjoyable”.

*Emotional Exhaustion.* We utilized the Chinese version of the emotional exhaustion scale (Li & Shi, 2003) to measure participants’ emotional exhaustion, which consists of five items. An example of the items included is, “Work makes me feel exhausted”.

*Stress mindset.* Crum et al. (2013) developed an eight-item scale to assess individuals’ beliefs about the effects of stress, with four of the items scored in reverse. An example item is “Experiencing stress facilitates my learning and growth”. This scale has also been validated for use in China (He et al., 2022).

*Control variables*. Active cyber incivility was measured using a revised Chinese version of the scale from Yan et al. (2023), similar to the passive cyber incivility assessment. The active cyber incivility scale consists of seven items, with an example as “Sending you messages in a rude and impolite tone”. Responses were recorded on a scale from 1 (never) to 5 (always occurs) (Table 1).

### 3.3. Analysis

Calculations for descriptive statistics, correlations, and exploratory factor analysis were performed using SPSS version 26.0. Confirmatory factor analysis (CFA) was conducted through Mplus 8.3 software. Regression analysis was performed using the PROCESS 4.0 (model 84) macro in SPSS 26.0 (Hayes, 2022). To mitigate multicollinearity, continuous variables were standardized (Luo & Jiang, 2012). Additionally, bootstrapping was conducted with 5000 samples, as recommended by Hayes (2022) to ensure that the confidence intervals for the indirect effects were reliably stable.

## 4. Results

### 4.1. Common Method Bias Check

Both Harman’s one-factor test and the CFA were employed to assess the potential influence of common method bias on the results (Tang & Wen, 2020). If an exploratory factor analysis shows that a single factor explains less than 40% of the total covariance observed among the variables, the existence of common method variance may not be statistically significant (Baumgartner et al., 2021). In the present investigation, a total of seven factors were identified by an unrotated exploratory factor analysis. Each of these factors illustrated an eigenvalue beyond the threshold of 1.0, indicating their significance. Moreover, the largest of the six remaining components represented 26.51% of the overall variance. After conducting CFA to assess the factorial validity of the five measures, the results indicated that the six-factor model fit our data well (see Table 2).

### 4.2. Descriptive Statistics

Table 3 provides a summary of the average values, variability measures, and relationships among the key variables examined in this study.

### 4.3. Hypothesis Testing

We used PROCESS Model 84 to analyze Hypotheses 1–5. The regression analysis results are displayed in Table 4.

After controlling for the effect of active cyber incivility, the direct impact of passive cyber incivility on work engagement was not found to be statistically significant (β = –0.04, *SE* = 0.05, *p* = 0.387). Therefore, Hypothesis 1 was not supported.

A negative relationship was observed between passive cyber incivility and intrinsic motivation (β = –0.17, *SE* = 0.07, *p* = 0.016, 95%CI [–0.31, –0.03]). Conversely, intrinsic motivation was found to positively predict work engagement (β = 0.43, *SE* = 0.04, *p* < 0.001, 95%CI [0.35, 0.51]). The findings indicate that passive cyber incivility had a negative indirect influence on worker engagement by intrinsic motivation (β = –0.07, *SE* = 0.03, 95%CI [–0.15, –0.01]. Hypothesis 2 was thus confirmed.

Subsequently, passive cyber incivility was identified as being positively correlated with emotional exhaustion (β = 0.22, *SE* = 0.06, *p* = 0.001, 95%CI [0.09, 0.34]), with emotional exhaustion also negatively predicting work engagement (β = –0.47, *SE* = 0.04, *p* < 0.001, 95%CI [–0.56, –0.39]). The mediated effect of passive cyber incivility on work engagement, through the pathway of intrinsic motivation, was found to be –0.10, *SE* = 0.03, 95%CI [–0.17, –0.04]. Hypothesis 3 was thus supported.

The serial mediation analysis revealed that passive cyber incivility had an impact on intrinsic motivation, which subsequently impacted emotional exhaustion. The final indirect effect on work engagement was –0.03, *SE* = 0.02, 95%CI [–0.06, –0.01], supporting Hypothesis 4.

Lastly, it was found that the moderating effect of stress mindset on the relationship between passive cyber incivility and emotional exhaustion was not statistically significant (β = 0.08, *SE* = 0.05, *p* = 0.080, 95%CI [–0.01, 0.17]). Similarly, the moderating effect of stress mindset on the relationship between passive cyber incivility and intrinsic motivation was not statistically significant (β = 0.05, *SE* = 0.05, *p* = 0.378). Therefore, Hypothesis 5a and 5b were not validated.

In addition, we found that the control variable, active cyber incivility, had a significant positive effect on work engagement (β = 0.11, *SE* = 0.05, 95% CI = [0.02, 0.20]) in the model analysis.

**Figure 2:**
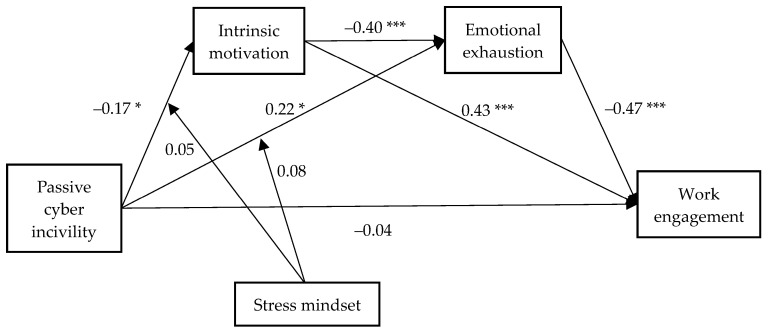
Summary of results. Note: *** *p* < 0.001 and * *p* < 0.05; active cyber incivility is controlled.

In conclusion, Hypotheses 1, 5a, and 5b were not confirmed, while Hypotheses 2 to 3 were validated. Figure 2 provides a summary of the results.

## 5. Discussion

### 5.1. Findings

This study explores the mechanism of passive cyber incivility on work engagement, focusing on the mediating roles of intrinsic motivation and emotional exhaustion, as well as the moderating role of stress mindset.

Firstly, this study reveals that the direct effect of PCI on work engagement is insignificant. This suggests that passive cyber incivility may not directly disrupt employees’ work engagement. Instead, its effect is more pronounced through indirect mechanisms. In contrast, passive cyber incivility significantly reduces employees’ intrinsic motivation, which in turn negatively affects work engagement. This finding points out that intrinsic motivation plays a crucial mediating role in the relationship between PCI and work engagement. These results align with Self-Determination Theory, which posits that intrinsic motivation is essential for sustaining positive work states, and that cyber incivility may undermine this motivational force (Ryan & Deci, 2020). Additionally, the Conservation of Resources theory supports this by suggesting that PCI, as a stressor, depletes the psychological resources essential for managing workplace demands, thereby reducing intrinsic motivation and affecting work engagement (Hobfoll, 1989).

Further, this study highlights that emotional exhaustion mediates the relationship between passive cyber incivility and work engagement. PCI increases emotional exhaustion, which then reduces work engagement. Emotional exhaustion is a key stress response that led to burnout and performance (Horvat & Tement, 2020). This result highlights the need to address emotional exhaustion in the workplace.

Moreover, the result shows a serial mediation effect, wherein passive cyber incivility negatively impacts intrinsic motivation, which subsequently increases emotional exhaustion, ultimately reducing work engagement. This finding emphasizes the complex psychological mechanisms through which cyber incivility gradually diminishes work engagement. It points out the importance to consider the interaction between employees’ psychological well-being and motivational states when addressing workplace incivility (Rode, 2016).

However, this study does not prove the moderating effect of stress mindset. This may be attributed to the complexity of stress mindset, as employees’ perceptions of stress may be influenced by various individual differences and contextual factors, potentially weakening its moderating effect (Keech et al., 2018). This finding opens new avenues for future research to investigate the dynamic nature of stress mindset and its role across various contexts.

Notably, an interesting and unexpected finding was that active cyber incivility, as a control variable, showed a positive effect on work engagement. This suggests that moderate levels of incivility may stimulate adaptive coping strategies, thereby enhancing work engagement. The finding challenges traditional perspectives and highlights the need for managers to holistically assess the behavioral dynamics within the work environment, rather than solely aiming to eliminate conflict (Zhou et al., 2022).

### 5.2. Limitations and Future Directions

This study has several limitations that should be considered in future research. Although a time-lagged design was used to reduce common method biases, the limitations of self-report measures remain a concern. Self-reporting may introduce biases stemming from social desirability or memory mistakes (Baumgartner et al., 2021). To improve accuracy, future studies should enhance the validity by combining mixed methods, or other objective measurements. For instance, using observational data or physiological measures could provide a more comprehensive understanding of PCI and its effects.

Another limitation is the time lag, which may not fully capture the long-term effects of PCI. Further research should use longer time lags to gather more detailed and realistic insights. Additionally, this study mainly focuses on the internal experiences of individuals, potentially neglecting the influence of external variables such as third-party perspectives or organizational factors. Future research should adopt broader methodologies by combining self-assessment with multiple data sources (Rosen et al., 2016). Conducting multi-level studies could provide a deeper understanding of how organizational factors interact with individual experiences of PCI, offering a more holistic view of its dynamics.

As the moderating effect of stress mindset, our findings diverged from our hypothesis. The subtle and ambiguous nature of PCI may not sufficient to activate the influence of a stress mindset. This suggests a potential ’threshold effect’, where only more severe instances of incivility might trigger the benefits of a positive stress mindset. Future research should explore varying levels of incivility and their differential impacts on employees, focusing on how these stressors interact with stress mindset. Additionally, workplace interventions should consider the nuanced nature of incivility, tailoring strategies to the specific intensity and context of these behaviors.

Moreover, the cultural setting of our study, conducted in China, may limit the generalizability of the findings to other cultural settings (Liu et al., 2021). Chinese culture, which emphasizes harmony over conflict, likely shapes employee responses to PCI in ways that differ from more individualistic cultures. Future research should consider cross-cultural comparisons to explore how PCI affects employees in different cultural settings. Such studies could uncover unique psychological patterns and broaden our understanding of workplace incivility across diverse environments.

### 5.3. Practical Implications

The findings of this study provide some practical advice for the workplace. Firstly, companies need to recognize PCI as a real problem and try to create a mutually respectful work environment. To cope with PCI, organizations should develop clear norms for online communication (Wang et al., 2024). These norms should include using correct grammar, expressing clear information, and responding to messages in a timely manner (Nag et al., 2022). This practice not only shows respect but also reduces misunderstandings in the workplace.

Secondly, organizations should provide training to help employees recognize uncivil behavior and learn how to avoid it (Giumetti et al., 2013). Also, implementing a policy of zero tolerance for PCI proves effective in reducing the occurrence of similar behaviors. In addition, enterprises should provide employees with meaningful work opportunities and platforms for skill development in order to stimulate intrinsic motivation. To manage emotional exhaustion, companies can offer stress management programs and organize daily healthy activities (Nicholson & Griffin, 2015).

As the study was conducted in China, employees may favor a stoic coping style over direct confrontation (Wong et al., 2016). Therefore, companies need to adapt measures to cultural characteristics. For instance, they should include cultural sensitivity training or adopt more flexible communication strategies to make their efforts more effective. Developing adaptive strategies to address differences in cultural contexts can improve the relevance and effectiveness of PCI management strategies.

## 6. Conclusions

Building on the COR theory, this research specifically aims to explore the impact of PCI on work engagement. Our findings suggest that this relationship is mediated by distinct motivational and emotional processes, rather than being directly influenced. Notably, the expected moderating role of stress mindset was not supported. Understanding insights into the effects of passive cyber incivility is essential for understanding how individuals navigate their professional and personal responsibilities in digital work environments. Furthermore, this study also highlights that there are several other matters that need to be examined when promoting stress management strategies in the workplace.

## Figures and Tables

**Table 1:** The results of Cronbach’s α, AVE, and CR.

Variables	Cronbach’s α	AVE	CR
1. Passive cyber incivility	0.82	0.53	0.87
2. Emotional exhaustion	0.91	0.74	0.93
3. Intrinsic motivation	0.90	0.66	0.92
4. Stress mindset	0.79	0.56	0.91
5. Work engagement	0.92	0.61	0.93
6. Active cyber incivility	0.89	0.61	0.92

**Table 2:** Results of confirmatory factor analysis.

Model	χ^2^	*df*	χ^2^/*df*	RMSEA	CFI	TLI	SRMR
Six-factor model (A, B, C, D, E, F)	1472.02	764	1.97 ***	0.06	0.90	0.89	0.07
Five-factor model (A + B, C, D, E, F)	1669.55	769	2.17 ***	0.06	0.87	0.86	0.07
Four-factor model (A + B, C + D, E, F)	2111.16	773	2.73 ***	0.08	0.80	0.79	0.09
Three-factor model (A + B, C + D + E, F)	2483.90	776	3.20 ***	0.09	0.75	0.74	0.09
Two-factor model (A + B + F, C + D + E)	3540.72	778	4.55 ***	0.11	0.60	0.57	0.15
One factor model (A + B + C + D + E + F)	4381.16	779	5.62 ***	0.12	0.47	0.44	0.15

Note(s): *** *p* < 0.001; A represents passive cyber incivility, B represents active cyber incivility, C represents stress mindset, D represents intrinsic motivation, E represents work engagement, and F represents emotional exhaustion; active cyber incivility is controlled.

**Table 3:** Means, standard deviations, and correlations among variables (N = 306).

Variable	M ± SD	1	2	3	4	5
1. Passive cyber incivility	2.61 ± 0.77	—				
2. Emotional exhaustion	3.71 ± 1.47	0.31 ***	—			
3. Intrinsic motivation	4.61 ± 1.26	−0.14 *	−0.46 ***	—		
4. Work engagement	5.10 ± 1.05	−0.18 **	−0.66 ***	−0.65 ***	—	
5. Stress mindset	4.37 ± 0.90	−0.13 *	−0.27 ***	0.21 ***	0.33 ***	—
6. Active cyber incivility	1.99 ± 0.78	0.61 ***	0.22 ***	−0.04	−0.03	−0.18 **

Note(s): two-tailed: *** *p* < 0.001, ** *p* < 0.01, and * *p* < 0.05; raw scores were used for the calculation of the correlation coefficients.

**Table 4:** Results of regression analysis (N = 306).

	Outcome 1 (Intrinsic Motivation)			Outcome 2 (Emotional Exhaustion)			Outcome 3 (Work Engagement)		
	β	*SE*	95%CI	β	*SE*	95%CI	β	*SE*	95%CI
Constant	0.01	0.06	[−0.10, 0.12]	0.01	0.05	[−0.09, 0.11]	0.001	0.04	[−0.07, 0.07]
Independent variable									
Passive cyber incivility	−0.17 *	0.07	[−0.31, −0.03]	0.22 **	0.06	[0.09, 0.34]	−0.04	0.05	[−0.13, 0.05]
Mediating variables									
Intrinsic motivation				−0.40 ***	0.05	[−0.50, −0.30]	0.43 ***	0.04	[0.35, 0.51]
Emotional exhaustion							−0.47 ***	0.04	[−0.56, −0.39]
Moderating variable									
Stress mindset	0.21 ***	0.06	[0.10, 0.32]	−0.14 **	0.05	[−0.24, −0.04]			
PCI × SM	0.05	0.05	[−0.06, 0.15]	0.08	0.05	[−0.01, 0.17]			
Control variable									
Active cyber incivility	0.11	0.07	[−0.03, 0.25]	0.05	0.06	[−0.07, 0.17]	0.11 *	0.05	[0.02, 0.20]
*R* ^2^	0.07			0.30			0.60		
*F*	5.34 ***			25.61 ***			111.15 ***		

Note(s): *** *p* < 0.001, ** *p* < 0.01, and * *p* < 0.05. PCI represents passive cyber incivility; SM represents stress mindset; active cyber incivility is controlled.

## Data Availability

The data presented in this study are available upon request from the corresponding author.

## Notes

1 Credamo is an online platform for survey, which offers professional participant recruitment and data collection services, ensuring a diverse and representative sample.

2 Note: Age Distribution:19–20 years old: 1%, 21–30 years old: 63.7%, 31–40 years old: 29.4%, 41–50 years old: 4.9%, 51 years and over: 1%; Organizational Venture Duration: Less than 2 years: 7.5%, 3–5 years: 36.9%, 6–10 years: 39.9%, 11–20 years: 13.1%, 21–30 years: 2.6%.
